# Supplementary material for: Chemistry in Fungal Bioluminescence: Theoretical Studies on Biosynthesis of Luciferin from Caffeic Acid and Regeneration of Caffeic Acid from Oxidized Luciferin
Source: J Fungi (Basel). 2023 Mar 18;9(3):369. doi: 10.3390/jof9030369 (PMC10053366; doi:10.3390/jof9030369)
Supplement: Supplementary file 1 [file jof-09-00369-s001.zip › jof-2240199-supplementary.pdf]

Supporting Information

Chemistry in Fungal Bioluminescence: Theoretical Studies on Biosynthesis of Luciferin from Caffeic Acid and Regeneration of Caffeic Acid from Oxidized Luciferin

Xiayu Liu[1], Mingyu Wang[2], Ya-Jun Liu[1,3,*]

1 Key Laboratory of Theoretical and Computational Photochemistry,

Ministry of Education, College of Chemistry, Beijing Normal University,

Beijing 100875, China,

2 School of Science, Hainan University, Haikou 570228, China

3 Center for Advanced Materials Research, Beijing Normal

University, Zhuhai 519087, China,

Table of contents

Detailed Simplified scheme of related molecules in Stage 1 2

References 4

The XYZ Cartesian Coordinates of optimized equilibrium structures (unit: Ångstrom) 5

Detailed Simplified scheme of related molecules in Stage 1

During the biosynthesis of hispidin, adenosine triphosphate-magnesium ion complex (Mg-ATP) and CoA are involved in the reaction, which contain many atoms and greatly raised the complexity of calculation. To solve this, we only reserved the key reaction positions in theoretical research, and the rest parts were simplified according to the principle that the pK_a values of substituted conjugated acids are similar. The most critical reaction site in MgATP is the first phosphate linked to adenosine, while magnesium pyrophosphate ion complex (Mg-PPi) plays the role of leaving group in the reaction. The Mg-PPi tends to exist in the form of $MgHP_2O_7^-$[1]. Because the pKa value of $MgH_2P_2O_7$ (pK_{a1}=4.4, pK_{a2}=6.6) is similar to that of CH_3COOH (pK_a=4.75), the part of Mg-PPi in Mg-ATP can be simplified to CH_3COO^-[2], as shown in Scheme S1. After the leaving of Mg-PPi, the remaining adenosine monophosphate (AMP) still acts as a leaving group and has a large number of atoms, which is not directly related to the reaction process. As the pK_a values of AMP (pK_{a1}=3.8, pK_{a2}=6.2)[3] and dihydromethyl phosphate (pK_{a1}=2.2, pK_{a2}=7.9)[2] are similar, adenosine can be simplified as methyl. Therefore, as shown in Scheme S2, in the quantum chemistry calculation, acetyl methyl phosphate, a simplified calculation model, is used to simulate the behavior of Mg-ATP in the actual reaction process.

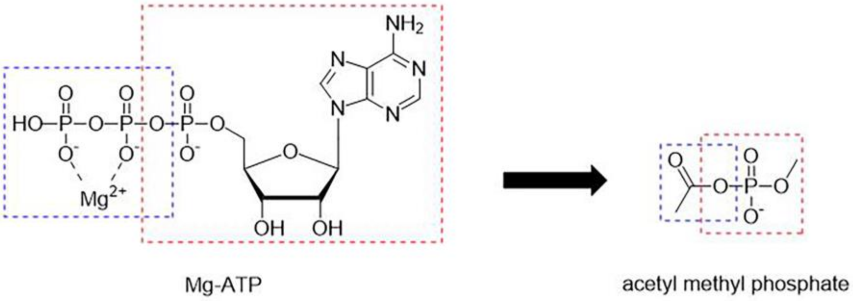

Scheme S1. Simplification strategy of the calculation model of MgATP

After CoA and caffeic acid are combined, caffeoyl-CoA achieves the growth of carbon chain through condensation reaction, which involves the process of transferring caffeoyl from CoA to cysteine. This progress is called loading of substrate. Whether CoA or cysteine, it is the sulfhydryl group in the molecule that actually involved in the reaction. The large volume group linked to sulfhydryl group in CoA and cysteine do not actually participate in the reaction process, and the pK_a values of sulfhydryl group in CoA and cysteine are 9.83 and 10.3, respectively[4, 5, 6, 7], while that of sulfhydryl group in methyl mercaptan is 10.3[3], so large groups in both molecules that link to sulfhydryl groups can be simplified as methyl, that is, CoA and cysteine could be simplified as methyl mercaptan. The pK_a value of sulfhydryl group has little change before and after the simplification of the model, which indicates that the simplification method is reasonable.

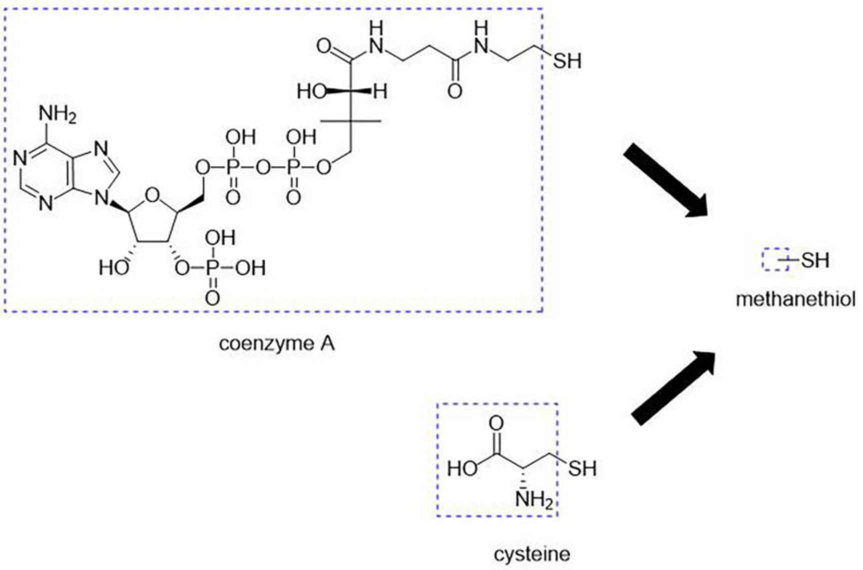

Scheme S2. Simplification strategy of the calculation model of CoA and cysteine.

The 3-hydoxylation process of hispidin involves flavin peroxide (FADH-4a-OOH), and the long phosphoribosyl side chain in its molecule is not directly involved in the process. Therefore, as shown in Scheme S3, this part is replaced by methyl in the calculation, that is, FADH-4a-OOH is simplified as peroxide of lumiflavin (LFOOH). The rationality of this simplified way has been confirmed by theoretical simulation of other flavins participating in the reaction,[8] and imidazole cation or neutral imidazole molecule is introduced to simulate the proton donor or proton acceptor near the reaction center. When studying the proton transfer process in substrate molecules assisted by hydrogen bond network in the environment, water molecules were introduced as water bridges.

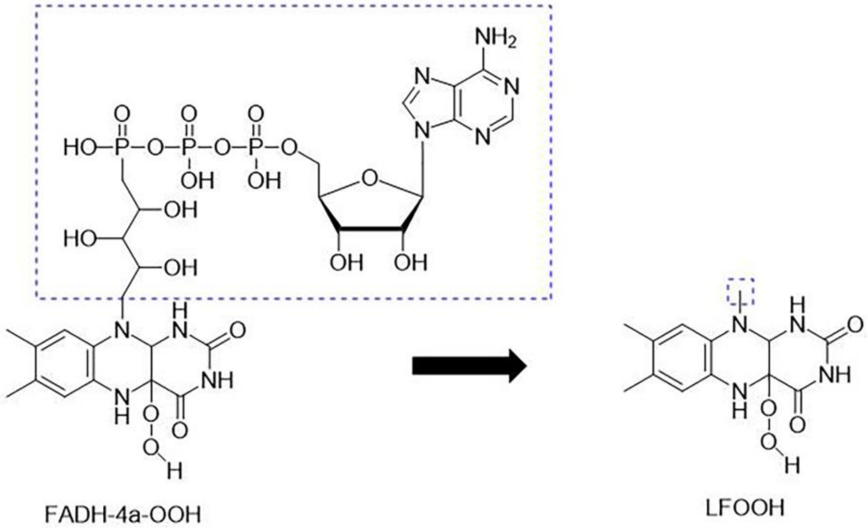

Scheme S3. Simplification strategy of the calculation model of flavin peroxide

References

1. Humberto Saint-Martin LER-V, Alejandro Ramı´rez-Solı´s, and Iva´n Ortega-Blake. Toward an Understanding of the Hydrolysis of Mg−PPi. An ab Initio Study of the Isomerization Reactions of Neutral and Anionic Mg−Pyrophosphate Complexes. *J Am Chem Soc*. **1996**, *118*, 12167-12173.
2. Smith P. A.S. Ionization-Constants of Inorganic Acids and Bases in Aqueous-Solution, 2nd Edition - PERRIN,DD. *J Am Chem Soc*. **1984**, *106*, 7298-7298.
3. Greenstein G.R. The Merck Index: An Encyclopedia of Chemicals, Drugs, and Biologicals (14th Edition). *Reference reviews*. **2007**, *21*, 40-40.
4. Gout I. Coenzyme A: a protective thiol in bacterial antioxidant defence. *Biochem Soc Trans*. **2019**, *47*, 469-476.
5. Marques S.M., da Silva JC. An optimized luciferase bioluminescent assay for coenzyme A. *Anal Bioanal Chem*. **2008**, *391*, 2161-2168.
6. David A. Keire E.S., Wei Guo, Béla NoszáV and Dallas L. Rabenstein. Kinetics and Equilibria of Thiol/Disulfide Interchange Reactions of,Selected Biological Thiols and Related Molecules with Oxidized Glutathione. *J Org Chem*. **1992**, *57*, 123-127.
7. David A. Keire J.M.R., and Dallas L. Rabenstein. Microscopic Protonation Equilibria and Solution Conformations of Coenzyme A and Coenzyme A Disulfides. *J. Org. Chem*. **1992**, *57*, 4427-4431.
8. Luo Y.L., Liu Y.J. Theoretical Insight into a Nonadiabatic Proton-Coupled Electron Transfer Mechanism of Reduced Flavin Oxygenation. *J Phys Chem A*. **2019**, *123*, 4354-4359.

The XYZ Cartesian Coordinates of optimized equilibrium structures (unit: Ångstrom)

$R_{1.1.1}$

C	3.096036000	1.522747000	0.254501000
C	2.115288000	2.263276000	-0.269844000
H	1.279752000	1.812364000	-0.798830000
C	2.100482000	3.778874000	-0.194320000
O	3.057476000	4.351089000	0.394947000
O	1.112687000	4.337516000	-0.743953000
H	3.900480000	2.063685000	0.749552000
C	3.239104000	0.059068000	0.210212000
C	4.452642000	-0.509079000	0.611447000
C	2.216624000	-0.794822000	-0.231189000
C	4.655886000	-1.886682000	0.559538000
H	5.251930000	0.135063000	0.964501000
C	2.415468000	-2.162998000	-0.290035000
H	1.245606000	-0.411229000	-0.523027000
C	3.641211000	-2.711768000	0.105094000
H	5.604878000	-2.315984000	0.870122000
P	-2.664702000	0.093750000	-0.448426000
O	-1.950327000	-1.181694000	-0.752918000
O	-3.803852000	-0.237576000	0.797570000
O	-1.690361000	1.019309000	0.451224000
O	-3.396988000	0.894657000	-1.481331000
C	-2.028579000	2.383788000	0.713755000
H	-2.991881000	2.451825000	1.228411000
H	-1.239587000	2.782972000	1.353544000
H	-2.059200000	2.957144000	-0.215293000
C	-4.882370000	-1.018078000	0.698304000
O	-5.628887000	-1.125439000	1.655878000
C	-5.119823000	-1.739224000	-0.601106000
H	-5.198460000	-1.018145000	-1.418484000
H	-4.267519000	-2.384911000	-0.824024000
H	-6.033188000	-2.326689000	-0.522121000
O	3.734302000	-4.080988000	0.008762000
H	4.587527000	-4.390031000	0.335946000
O	1.412292000	-2.974229000	-0.729909000
H	1.707626000	-3.894068000	-0.674234000

$TS_{1.1.1}$

C	1.926949000	1.763886000	-0.209797000

C	0.628652000	1.515844000	-0.408367000
H	0.235185000	0.513580000	-0.529690000
C	-0.386586000	2.624900000	-0.454762000
O	0.004493000	3.807385000	-0.536148000
O	-1.625623000	2.296238000	-0.398613000
H	2.224089000	2.804790000	-0.097984000
C	3.014433000	0.778023000	-0.110000000
C	4.307244000	1.224944000	0.181078000
C	2.811084000	-0.599197000	-0.288907000
C	5.370782000	0.331822000	0.297637000
H	4.485509000	2.286225000	0.322237000
C	3.863200000	-1.489260000	-0.174058000
H	1.827649000	-0.994427000	-0.516592000
C	5.149296000	-1.023263000	0.122018000
H	6.370680000	0.691262000	0.526068000
P	-2.542066000	0.435123000	-0.125459000
O	-1.826338000	-0.221360000	-1.260074000
O	-3.403896000	-1.393118000	0.575690000
O	-1.720515000	0.326302000	1.267514000
O	-3.867964000	1.120077000	-0.086916000
C	-2.356415000	0.745197000	2.467407000
H	-3.267001000	0.165503000	2.636728000
H	-1.645642000	0.562764000	3.276026000
H	-2.598071000	1.811324000	2.426234000
C	-4.250397000	-2.139094000	-0.027177000
O	-4.771761000	-3.148861000	0.496289000
C	-4.626283000	-1.771392000	-1.453934000
H	-5.040263000	-0.760145000	-1.470678000
H	-3.724779000	-1.749752000	-2.070736000
H	-5.348450000	-2.483171000	-1.856376000
O	6.120312000	-1.992505000	0.213583000
H	6.971014000	-1.605635000	0.453115000
O	3.647076000	-2.825174000	-0.351478000
H	4.483052000	-3.295497000	-0.222075000

$P_{1.1.1}$

C	-2.206308000	-0.772330000	1.317192000
C	-1.191133000	-1.276137000	0.604251000
H	-1.086314000	-1.129832000	-0.463278000
C	-0.152235000	-2.087515000	1.280459000
O	-0.240251000	-2.451668000	2.442417000
O	0.935633000	-2.438815000	0.579634000
H	-2.219789000	-0.984828000	2.383758000
C	-3.314476000	0.048808000	0.821115000

C	-4.263517000	0.513986000	1.736156000
C	-3.461099000	0.387901000	-0.533256000
C	-5.335087000	1.300153000	1.320634000
H	-4.163137000	0.261917000	2.786829000
C	-4.523736000	1.166765000	-0.949982000
H	-2.747257000	0.053537000	-1.277825000
C	-5.465028000	1.625824000	-0.018817000
H	-6.067214000	1.658992000	2.038432000
P	1.413016000	-2.005483000	-1.004397000
O	0.263941000	-2.242838000	-1.931898000
O	5.555008000	2.766272000	0.502787000
O	1.556703000	-0.394773000	-0.825984000
O	2.744394000	-2.666218000	-1.147806000
C	2.620737000	0.146461000	-0.035775000
H	3.586869000	-0.243581000	-0.360719000
H	2.601258000	1.227840000	-0.169944000
H	2.471299000	-0.091770000	1.021684000
C	6.744985000	2.392299000	0.317677000
O	7.783816000	3.077752000	0.523522000
C	6.933569000	0.969559000	-0.230515000
H	6.343450000	0.257564000	0.354941000
H	6.556493000	0.926913000	-1.257815000
H	7.983214000	0.669109000	-0.225078000
O	-6.476051000	2.389748000	-0.540204000
H	-7.069877000	2.702407000	0.153405000
O	-4.656183000	1.489594000	-2.267111000
H	-5.440351000	2.046889000	-2.376116000

$R_{1.1.2}$

C	-5.028259000	-0.846475000	1.232521000
H	-4.278833000	-0.634323000	1.986329000
C	-6.202708000	-1.481079000	1.613179000
H	-6.376815000	-1.764447000	2.645461000
C	-7.180638000	-1.766352000	0.668375000
O	-8.329055000	-2.388178000	1.044284000
H	-8.888610000	-2.514091000	0.263308000
C	-6.973691000	-1.409085000	-0.667167000
O	-7.996872000	-1.731564000	-1.520310000
H	-7.787758000	-1.484500000	-2.429666000
C	-5.804017000	-0.775686000	-1.046549000
H	-5.656172000	-0.502593000	-2.088091000
C	-4.808336000	-0.483812000	-0.102015000
C	-3.592275000	0.185815000	-0.564542000
H	-3.556858000	0.424385000	-1.625700000

C	-2.522912000	0.534115000	0.163436000
H	-2.439507000	0.345033000	1.227259000
C	-1.376151000	1.206847000	-0.478390000
O	-1.301738000	1.489496000	-1.656169000
S	8.034546000	-3.118271000	-0.260967000
C	9.173031000	-1.763968000	-0.758685000
H	8.682936000	-0.786805000	-0.687993000
H	10.058482000	-1.738119000	-0.117366000
H	9.510091000	-1.891718000	-1.790992000
O	-0.414815000	1.482879000	0.429547000
P	1.129440000	2.076714000	0.091110000
O	1.751189000	2.238111000	1.440610000
O	1.767491000	1.195909000	-0.951743000
O	0.811827000	3.498810000	-0.603722000
C	0.278172000	4.565633000	0.182941000
H	-0.744889000	4.337705000	0.498228000
H	0.899728000	4.746200000	1.062848000
H	0.269735000	5.449527000	-0.455081000
H	6.500495000	-1.953719000	0.117492000
N	5.690214000	-1.249478000	0.302611000
C	4.755455000	-0.908378000	-0.577298000
H	4.683477000	-1.282500000	-1.586352000
N	3.925283000	-0.034546000	-0.012922000
H	3.078213000	0.422428000	-0.452348000
C	4.340454000	0.194631000	1.277330000
H	3.799166000	0.877513000	1.914108000
C	5.451144000	-0.570979000	1.473841000
H	6.084489000	-0.691232000	2.337729000

$TS1_{1.1.2}$

C	3.712865000	-0.291374000	-1.137687000
H	3.063356000	-0.307322000	-2.005552000
C	5.088139000	-0.209882000	-1.325433000
H	5.512537000	-0.151472000	-2.322193000
C	5.945450000	-0.212568000	-0.234127000
O	7.296331000	-0.134030000	-0.420130000
H	7.728781000	-0.149974000	0.446139000
C	5.411437000	-0.304667000	1.052142000
O	6.333532000	-0.307258000	2.070858000
H	5.898173000	-0.355652000	2.930862000
C	4.041958000	-0.387471000	1.240109000
H	3.645018000	-0.456975000	2.249921000
C	3.162401000	-0.372467000	0.147678000
C	1.717324000	-0.443172000	0.403548000

H	1.418209000	-0.731001000	1.408926000
C	0.741496000	-0.169994000	-0.469336000
H	0.934258000	0.151718000	-1.486135000
C	-0.690635000	-0.295612000	-0.090085000
O	-1.076152000	-0.614782000	1.031156000
O	-1.481851000	0.657261000	-0.858268000
P	-1.981505000	2.079507000	-0.293935000
O	-2.503654000	2.887692000	-1.429409000
O	-2.924810000	1.854173000	0.931287000
O	-0.717046000	2.761158000	0.437541000
C	0.261655000	3.447330000	-0.349797000
H	0.841117000	2.738735000	-0.947511000
H	-0.216153000	4.179373000	-1.004228000
H	0.924734000	3.951396000	0.353167000
S	-1.109891000	-2.085412000	-1.557054000
C	-0.466121000	-3.319625000	-0.386644000
H	0.620789000	-3.419886000	-0.461588000
H	-0.717228000	-3.006672000	0.632664000
H	-0.916018000	-4.298293000	-0.572639000
N	-4.581418000	-0.100684000	0.783920000
H	-3.603473000	1.049831000	0.879864000
C	-5.894366000	-0.224452000	1.178068000
H	-6.410421000	0.571460000	1.693389000
C	-6.363038000	-1.452982000	0.812766000
H	-7.322812000	-1.927569000	0.938346000
N	-5.306920000	-2.075000000	0.185079000
H	-5.308205000	-2.999667000	-0.219426000
C	-4.254099000	-1.230133000	0.187976000
H	-3.291061000	-1.465564000	-0.254470000

$IM_{1.1.2}$

C	3.715747000	-0.251995000	-1.133958000
H	3.125426000	-0.205370000	-2.042402000
C	5.095891000	-0.101693000	-1.217503000
H	5.581862000	0.069508000	-2.172421000
C	5.878821000	-0.176280000	-0.074003000
O	7.234806000	-0.030086000	-0.156472000
H	7.607347000	-0.102644000	0.734406000
C	5.265561000	-0.409607000	1.157543000
O	6.117592000	-0.474859000	2.233756000
H	5.626803000	-0.594853000	3.055998000
C	3.891383000	-0.562157000	1.240686000
H	3.430888000	-0.741506000	2.209229000
C	3.085863000	-0.477307000	0.096204000

C	1.630445000	-0.632939000	0.246531000
H	1.281762000	-1.014617000	1.203935000
C	0.693274000	-0.322757000	-0.650493000
H	0.931899000	0.086215000	-1.627639000
C	-0.770076000	-0.576090000	-0.382002000
O	-1.143408000	-0.909684000	0.770723000
O	-1.495664000	0.673898000	-0.996722000
P	-1.766566000	2.070925000	-0.300746000
O	-2.233951000	3.067446000	-1.309843000
O	-2.684933000	1.886098000	0.963833000
O	-0.407562000	2.548471000	0.436492000
C	0.596822000	3.215976000	-0.327781000
H	1.070107000	2.527311000	-1.033288000
H	0.169089000	4.061725000	-0.871245000
H	1.345172000	3.569690000	0.382045000
S	-1.340188000	-1.810884000	-1.802583000
C	-0.733534000	-3.328471000	-1.016930000
H	0.358107000	-3.382581000	-1.033628000
H	-1.076363000	-3.361761000	0.019573000
H	-1.138971000	-4.184397000	-1.560492000
N	-4.572935000	0.087847000	0.932206000
H	-3.448223000	1.195167000	0.913425000
C	-5.949049000	0.071565000	0.942633000
H	-6.528832000	0.981238000	0.987207000
C	-6.389312000	-1.219275000	0.883977000
H	-7.379082000	-1.646553000	0.868629000
N	-5.249689000	-1.992341000	0.838384000
H	-5.212248000	-2.998360000	0.772974000
C	-4.179086000	-1.168145000	0.866840000
H	-3.136423000	-1.471701000	0.827495000

$TS2_{1.1.2}$

C	3.702321000	-0.208239000	-1.118092000
H	3.105504000	-0.127762000	-2.019780000
C	5.078529000	-0.029127000	-1.203460000
H	5.554383000	0.195339000	-2.152336000
C	5.870833000	-0.139976000	-0.069132000
O	7.222555000	0.034503000	-0.154049000
H	7.603412000	-0.069055000	0.730310000
C	5.270746000	-0.436704000	1.155276000
O	6.131377000	-0.531578000	2.221954000
H	5.649001000	-0.689588000	3.042773000
C	3.900306000	-0.618775000	1.239889000
H	3.449993000	-0.847785000	2.202658000

C	3.086022000	-0.500860000	0.104709000
C	1.635632000	-0.692735000	0.258954000
H	1.301857000	-1.067341000	1.224299000
C	0.687818000	-0.428521000	-0.641996000
H	0.905898000	-0.038221000	-1.630115000
C	-0.757865000	-0.722757000	-0.355633000
O	-1.135043000	-1.009877000	0.793786000
O	-1.494297000	0.686464000	-1.013770000
P	-1.704658000	2.078098000	-0.331131000
O	-2.133570000	3.130005000	-1.306363000
O	-2.629579000	1.932930000	0.946625000
O	-0.330435000	2.507170000	0.424322000
C	0.700275000	3.132577000	-0.335298000
H	1.126033000	2.438100000	-1.065948000
H	0.316787000	4.015569000	-0.852736000
H	1.478327000	3.425253000	0.371060000
S	-1.436032000	-1.820850000	-1.769411000
C	-0.825295000	-3.407029000	-1.130701000
H	0.266976000	-3.430690000	-1.100480000
H	-1.215677000	-3.593952000	-0.128586000
H	-1.175198000	-4.193015000	-1.803162000
N	-4.593459000	0.169108000	0.937665000
H	-3.403987000	1.272749000	0.893917000
C	-5.969618000	0.177730000	0.941220000
H	-6.532625000	1.098744000	0.963515000
C	-6.435276000	-1.105441000	0.907875000
H	-7.433035000	-1.513958000	0.896212000
N	-5.310900000	-1.901007000	0.885000000
H	-5.292423000	-2.908719000	0.841580000
C	-4.225140000	-1.095098000	0.901751000
H	-3.192124000	-1.427456000	0.873487000

$P_{1.1.2}$

C	1.003355000	3.411103000	0.369191000
H	2.025915000	3.753242000	0.483278000
C	-0.033310000	4.311827000	0.566528000
H	0.161894000	5.345990000	0.829253000
C	-1.350590000	3.887862000	0.436395000
O	-2.373915000	4.757326000	0.636096000
H	-3.202012000	4.265652000	0.508578000
C	-1.638062000	2.556330000	0.102593000
O	-2.957876000	2.260692000	0.002575000
H	-3.136356000	1.280825000	-0.014259000
C	-0.603099000	1.660833000	-0.108804000

H	-0.847949000	0.642355000	-0.405757000
C	0.733559000	2.077244000	0.030471000
C	1.778042000	1.085309000	-0.174589000
H	1.422639000	0.084496000	-0.402949000
C	3.113248000	1.256841000	-0.110025000
H	3.563179000	2.224440000	0.086686000
C	4.025066000	0.134476000	-0.316160000
O	3.697479000	-1.039417000	-0.460811000
O	-1.946800000	-1.031480000	-1.468750000
P	-3.292972000	-1.453267000	-0.941645000
O	-4.502599000	-1.590980000	-1.817858000
O	-3.173179000	-2.785538000	-0.033005000
O	-3.630634000	-0.279183000	0.215676000
C	-4.865952000	-0.366125000	0.924520000
H	-5.709771000	-0.323396000	0.231420000
H	-4.908462000	-1.298976000	1.493670000
H	-4.906066000	0.481091000	1.611917000
S	5.738774000	0.614153000	-0.342921000
C	6.523754000	-0.993176000	-0.608958000
H	6.279476000	-1.676680000	0.203754000
H	6.203386000	-1.421746000	-1.558138000
H	7.598998000	-0.813109000	-0.629281000
N	-0.593595000	-3.251730000	0.791368000
H	-2.254477000	-2.963787000	0.317644000
C	0.165275000	-3.897793000	1.739666000
H	-0.277956000	-4.561277000	2.467689000
C	1.481830000	-3.558560000	1.586629000
H	2.369547000	-3.847899000	2.126564000
N	1.519928000	-2.692062000	0.520009000
H	2.334788000	-2.197618000	0.155697000
C	0.253395000	-2.536939000	0.076193000
H	-0.045774000	-1.901244000	-0.747276000

$R_{1.2.1}$

C	2.691881000	-1.182815000	-0.216874000
H	2.591974000	-1.814515000	-1.097263000
C	4.016314000	-0.614893000	0.011517000
C	5.019345000	-0.869525000	-0.938031000
C	4.335796000	0.166001000	1.129983000
C	6.292068000	-0.355589000	-0.773143000
H	4.794479000	-1.475219000	-1.811895000
C	5.612225000	0.683683000	1.294013000
H	3.587705000	0.369698000	1.887537000
C	6.596104000	0.428027000	0.346037000

H	5.866616000	1.286119000	2.159003000
O	7.842275000	0.934397000	0.510938000
H	8.400687000	0.651798000	-0.229337000
O	7.331691000	-0.547352000	-1.641819000
H	7.084148000	-1.128998000	-2.371414000
C	1.586200000	-1.015151000	0.528524000
H	1.572806000	-0.403034000	1.424710000
C	0.331975000	-1.679353000	0.147459000
O	0.192072000	-2.430564000	-0.797530000
C	-4.487511000	-0.174598000	-2.062223000
H	-4.435681000	-1.145044000	-2.540892000
H	-3.997278000	0.667825000	-2.540581000
C	-5.217211000	0.018730000	-0.926939000
O	-5.330351000	1.074675000	-0.217475000
S	-1.031590000	-1.281330000	1.247855000
C	-2.374379000	-2.239622000	0.498245000
H	-3.130103000	-2.380784000	1.270548000
H	-2.812033000	-1.708569000	-0.347729000
H	-1.982840000	-3.205162000	0.179613000
S	-6.199290000	-1.452521000	-0.385859000
C	-6.447408000	-1.072449000	1.367964000
H	-6.554616000	0.008453000	1.468579000
H	-7.361238000	-1.568409000	1.698370000
H	-5.611223000	-1.414973000	1.982382000
C	-1.383876000	4.094875000	-0.163366000
C	-0.739305000	2.972563000	0.240588000
N	-1.688931000	1.972343000	0.298422000
C	-2.881153000	2.446795000	-0.055747000
N	-2.706366000	3.738343000	-0.338477000
H	0.296012000	2.801880000	0.484827000
H	-3.847673000	1.887606000	-0.116326000
H	-1.024969000	5.095198000	-0.339125000
H	-1.516280000	1.001229000	0.567471000
H	-3.451223000	4.352394000	-0.640987000

$TS_{1.2.1}$

C	1.810640000	-1.111917000	0.389978000
H	1.857594000	-2.178517000	0.598034000
C	3.078547000	-0.469007000	0.023855000
C	4.262601000	-1.212246000	0.138458000
C	3.169755000	0.848437000	-0.441874000
C	5.486502000	-0.653857000	-0.188896000
H	4.221470000	-2.240027000	0.489838000
C	4.397910000	1.409088000	-0.770518000

H	2.273030000	1.445673000	-0.562629000
C	5.562862000	0.664217000	-0.645480000
H	4.467909000	2.427984000	-1.136204000
O	6.764891000	1.215286000	-0.971312000
H	7.459726000	0.554403000	-0.833754000
O	6.687111000	-1.310210000	-0.102185000
H	6.569700000	-2.217850000	0.204656000
C	0.611972000	-0.525322000	0.498193000
H	0.471561000	0.536060000	0.328114000
C	-0.594328000	-1.306550000	0.878885000
O	-0.570694000	-2.513583000	1.093327000
C	-1.493014000	-0.931863000	-1.202893000
H	-0.880115000	-1.759700000	-1.540449000
H	-1.140560000	0.060748000	-1.469242000
C	-2.889746000	-1.101563000	-1.199437000
O	-3.758128000	-0.203981000	-1.106659000
S	-1.875267000	-0.296419000	1.796629000
C	-1.183608000	-0.469212000	3.472091000
H	-1.864376000	0.032163000	4.161963000
H	-1.125663000	-1.525748000	3.735420000
H	-0.194221000	-0.014367000	3.547228000
S	-3.439135000	-2.823434000	-1.274027000
C	-5.217737000	-2.620896000	-1.008348000
H	-5.651607000	-1.977610000	-1.773449000
H	-5.658504000	-3.617182000	-1.072179000
H	-5.415895000	-2.197080000	-0.023769000
C	-2.517057000	4.697540000	-0.227461000
C	-1.896113000	3.980777000	0.744057000
N	-2.218711000	2.658574000	0.527399000
C	-3.009547000	2.552792000	-0.533865000
N	-3.200394000	3.784916000	-1.006489000
H	-1.263843000	4.296249000	1.557455000
H	-3.395150000	1.598069000	-0.916279000
H	-2.533046000	5.755577000	-0.429700000
H	-1.931331000	1.828427000	1.077356000
H	-3.765783000	4.003349000	-1.816259000

$P_{1.2.1}$

C	0.965884000	-1.779935000	-1.021019000
H	1.117133000	-2.835062000	-1.242407000
C	2.148075000	-1.049290000	-0.581531000
C	3.364772000	-1.747149000	-0.499801000
C	2.125225000	0.309216000	-0.235885000
C	4.517981000	-1.105293000	-0.089403000

H	3.400789000	-2.801439000	-0.761604000
C	3.284512000	0.949359000	0.178695000
H	1.200386000	0.874718000	-0.288100000
C	4.484029000	0.250885000	0.253502000
H	3.277722000	1.998764000	0.452626000
O	5.613212000	0.884435000	0.657625000
H	6.348869000	0.253454000	0.654352000
O	5.748103000	-1.698486000	0.019154000
H	5.716705000	-2.628842000	-0.235851000
C	-0.284827000	-1.311519000	-1.187205000
H	-0.541965000	-0.272375000	-1.000086000
C	-1.350613000	-2.213399000	-1.650011000
O	-1.170736000	-3.397696000	-1.901597000
C	-2.734937000	-1.601407000	-1.849010000
H	-3.394844000	-2.385048000	-2.228433000
H	-2.674717000	-0.795723000	-2.586011000
C	-3.342610000	-1.001040000	-0.593654000
O	-3.720949000	0.151593000	-0.532567000
S	-0.876050000	1.282738000	2.867070000
C	-0.088776000	-0.361631000	2.810571000
H	0.955627000	-0.286573000	2.508091000
H	-0.134900000	-0.768522000	3.821509000
H	-0.621492000	-1.032414000	2.135829000
S	-3.482828000	-2.122222000	0.767854000
C	-4.203851000	-1.022263000	2.012491000
H	-5.151678000	-0.619916000	1.656426000
H	-4.371690000	-1.632942000	2.900085000
H	-3.514494000	-0.210309000	2.246015000
C	-0.640365000	4.570833000	-1.458220000
C	0.119819000	3.661817000	-0.777212000
N	-0.626643000	2.541873000	-0.481807000
C	-1.824361000	2.773409000	-0.977609000
N	-1.879376000	3.987518000	-1.578297000
H	1.155449000	3.749012000	-0.482722000
H	-2.668496000	2.099514000	-0.925782000
H	-0.420697000	5.549090000	-1.855223000
H	-0.701345000	1.626926000	1.571592000
H	-2.688976000	4.389367000	-2.027476000

$R_{1.2.2}$

C	-2.019571000	1.634541000	0.084219000
H	-2.366905000	2.612419000	0.412431000
C	-3.049249000	0.724958000	-0.412589000
C	-4.382156000	1.165991000	-0.418458000

C	-2.770159000	-0.564464000	-0.884993000
C	-5.394494000	0.346246000	-0.882988000
H	-4.623025000	2.161519000	-0.054657000
C	-3.786903000	-1.388017000	-1.347378000
H	-1.750354000	-0.931426000	-0.900204000
C	-5.102463000	-0.939761000	-1.349318000
H	-3.575315000	-2.385030000	-1.718043000
O	-6.093908000	-1.747485000	-1.803991000
H	-6.937692000	-1.273755000	-1.749847000
O	-6.719221000	0.691441000	-0.927775000
H	-6.855083000	1.607416000	-0.655281000
C	-0.701456000	1.396384000	0.183349000
H	-0.255290000	0.456856000	-0.118838000
C	0.209401000	2.419492000	0.722603000
O	-0.160615000	3.534761000	1.065391000
C	1.673937000	2.030548000	0.887366000
H	1.719078000	1.097905000	1.467931000
H	2.177720000	2.820848000	1.444955000
C	2.374482000	1.765393000	-0.430197000
O	1.953423000	1.007479000	-1.279541000
S	3.896563000	2.654171000	-0.616121000
C	4.439290000	2.024309000	-2.223203000
H	3.709116000	2.270339000	-2.993728000
H	5.388758000	2.512520000	-2.444548000
H	4.579972000	0.944627000	-2.175323000
C	0.318697000	-0.105497000	3.987442000
C	-0.956978000	-0.591428000	4.069060000
N	-1.068334000	-1.488252000	3.033689000
C	0.118297000	-1.508613000	2.376940000
N	0.983128000	-0.683045000	2.927987000
H	-1.770067000	-0.387426000	4.747477000
H	0.307626000	-2.128747000	1.510683000
H	0.794766000	0.624775000	4.625650000
H	-1.887115000	-2.027960000	2.794790000
H	3.088285000	-1.966080000	1.936000000
C	3.534667000	-2.704055000	1.261928000
H	4.615702000	-2.563323000	1.225030000
H	3.308817000	-3.698993000	1.654426000
C	2.886060000	-2.559259000	-0.088293000
O	1.692268000	-2.705181000	-0.268885000
S	3.999413000	-2.151971000	-1.407122000
C	2.834618000	-1.979961000	-2.781178000
H	2.192649000	-1.115378000	-2.613221000
H	3.432062000	-1.831770000	-3.681423000

H	2.235406000	-2.884426000	-2.881568000

TS$_{1.2.2}$

C	-2.826872000	-0.470476000	-0.883081000
H	-2.697765000	-1.177276000	-1.700870000
C	-4.203180000	-0.247213000	-0.445805000
C	-5.233924000	-0.921229000	-1.119817000
C	-4.543266000	0.604717000	0.613056000
C	-6.554004000	-0.745164000	-0.747088000
H	-4.993561000	-1.588456000	-1.943526000
C	-5.868005000	0.781079000	0.986561000
H	-3.769644000	1.135578000	1.155616000
C	-6.879697000	0.109508000	0.310802000
H	-6.135956000	1.438959000	1.806019000
O	-8.174964000	0.284052000	0.679390000
H	-8.741193000	-0.255343000	0.107111000
O	-7.622950000	-1.359331000	-1.345861000
H	-7.340651000	-1.936435000	-2.066151000
C	-1.710888000	0.095719000	-0.395759000
H	-1.724611000	0.810068000	0.419554000
C	-0.390438000	-0.238116000	-0.959547000
O	-0.239008000	-1.063702000	-1.854557000
C	0.813509000	0.516907000	-0.417007000
H	0.672172000	1.585151000	-0.614127000
H	1.698424000	0.173402000	-0.951394000
C	0.980980000	0.375833000	1.090896000
O	0.269038000	0.971242000	1.894111000
S	1.468004000	-1.392581000	1.509557000
C	1.632546000	-1.234553000	3.302554000
H	0.819517000	-0.602423000	3.661714000
H	1.548852000	-2.229069000	3.741351000
H	2.591342000	-0.789355000	3.573408000
C	4.841223000	-3.393544000	-0.168956000
C	5.925290000	-3.230164000	-0.968953000
N	5.962209000	-1.890876000	-1.301831000
C	4.940884000	-1.251766000	-0.730639000
N	4.253945000	-2.153488000	-0.038520000
H	6.658597000	-3.934454000	-1.324950000
H	4.688720000	-0.184309000	-0.799591000
H	4.442308000	-4.273065000	0.308792000
H	6.652545000	-1.445895000	-1.892058000
H	3.398138000	-1.931203000	0.500258000
C	2.989126000	1.362704000	1.351287000
H	2.613592000	1.972465000	2.164683000

H	3.610743000	0.520282000	1.638800000
C	3.354660000	2.009506000	0.160153000
O	4.008982000	1.526428000	-0.792242000
S	2.681461000	3.685596000	-0.025925000
C	3.145060000	4.050432000	-1.737483000
H	2.628639000	3.388556000	-2.433089000
H	2.847228000	5.082726000	-1.928161000
H	4.221254000	3.947816000	-1.872822000

$P_{1.2.2}$

C	-2.155873000	-0.333925000	-0.994419000
H	-1.632906000	-1.216610000	-1.357722000
C	-3.528808000	-0.545630000	-0.551236000
C	-4.045360000	-1.851078000	-0.588834000
C	-4.356073000	0.488599000	-0.093477000
C	-5.340417000	-2.108178000	-0.180433000
H	-3.420769000	-2.668970000	-0.937944000
C	-5.656475000	0.230527000	0.313835000
H	-3.986153000	1.506374000	-0.051677000
C	-6.156062000	-1.065992000	0.273742000
H	-6.300703000	1.026430000	0.670701000
O	-7.427888000	-1.314727000	0.673205000
H	-7.603839000	-2.264754000	0.596176000
O	-5.929092000	-3.344834000	-0.182247000
H	-5.306697000	-4.031908000	-0.450953000
C	-1.462972000	0.818769000	-1.002767000
H	-1.885330000	1.750568000	-0.642557000
C	-0.079283000	0.856693000	-1.491960000
O	0.495203000	-0.110034000	-1.976888000
C	0.641520000	2.203897000	-1.415031000
H	0.105869000	2.908560000	-2.058290000
H	1.655123000	2.053478000	-1.783088000
C	0.645191000	2.797096000	-0.019071000
O	-0.192910000	3.614155000	0.311272000
S	1.369502000	-1.011915000	2.409420000
C	2.967572000	-0.601722000	3.213056000
H	3.201643000	0.463055000	3.115172000
H	2.936017000	-0.837499000	4.279427000
H	3.793308000	-1.168221000	2.770372000
C	2.691795000	-3.568217000	-0.396323000
C	3.285418000	-3.621280000	-1.618476000
N	3.511300000	-2.311606000	-1.989764000
C	3.065227000	-1.498878000	-1.025682000
N	2.569913000	-2.242071000	-0.052360000

H	3.562333000	-4.453399000	-2.244371000
H	3.098939000	-0.418202000	-1.035137000
H	2.348746000	-4.358285000	0.251924000
H	3.930759000	-2.003199000	-2.855986000
H	2.095710000	-1.821672000	0.864368000
C	1.670357000	2.320431000	0.996101000
H	1.550275000	2.931993000	1.891195000
H	1.433730000	1.269299000	1.266182000
C	3.101521000	2.329771000	0.511707000
O	3.514164000	1.620822000	-0.388958000
S	4.143540000	3.462458000	1.377803000
C	5.724236000	3.145532000	0.556926000
H	5.650024000	3.361192000	-0.508518000
H	6.452434000	3.812642000	1.018849000
H	6.027919000	2.109557000	0.705440000

$R_{1.3.1}$

C	1.912872000	-0.565944000	0.039704000
H	1.664857000	-1.386284000	0.710883000
C	3.319675000	-0.179651000	0.000488000
C	4.224833000	-0.891120000	0.804416000
C	3.809272000	0.865025000	-0.794310000
C	5.568223000	-0.565157000	0.812321000
H	3.867294000	-1.706582000	1.427206000
C	5.157984000	1.189880000	-0.788858000
H	3.135491000	1.435330000	-1.423215000
C	6.044176000	0.479376000	0.012333000
H	5.542588000	1.998022000	-1.401183000
O	7.361639000	0.801665000	0.016498000
H	7.829503000	0.214271000	0.629301000
O	6.521558000	-1.199358000	1.563276000
H	6.134826000	-1.885469000	2.121574000
C	0.890140000	-0.031421000	-0.648705000
H	1.023081000	0.792304000	-1.342636000
C	-0.476656000	-0.547120000	-0.475324000
O	-0.761868000	-1.459696000	0.291606000
C	-1.555352000	0.130573000	-1.300531000
C	-2.918105000	-0.473667000	-1.057859000
O	-3.822115000	0.171838000	-0.562978000
C	-3.144480000	-1.912930000	-1.489352000
C	-3.329369000	-2.925120000	-0.364894000
O	-2.810422000	-4.018996000	-0.420956000
H	-4.062607000	-1.926770000	-2.087898000
H	-2.323307000	-2.286141000	-2.101206000

H	-1.294058000	0.032478000	-2.362512000
S	-4.411062000	-2.462320000	0.962020000
C	-4.331948000	-3.985017000	1.938267000
H	-4.687772000	-4.834889000	1.356675000
H	-3.311802000	-4.170238000	2.273283000
H	-4.980920000	-3.828384000	2.800590000
C	-1.405778000	4.751412000	0.048589000
C	-2.371703000	5.345326000	0.813229000
N	-3.361399000	4.399984000	0.942278000
C	-2.964048000	3.293482000	0.264940000
N	-1.783763000	3.470901000	-0.289168000
H	-2.443276000	6.322459000	1.263961000
H	-3.555914000	2.389915000	0.201948000
H	-0.463936000	5.169135000	-0.276290000
H	-1.570048000	1.201638000	-1.065570000
H	-4.227089000	4.504651000	1.450286000

$TS1_{1.3.1}$

C	2.094680000	-0.637656000	-0.193749000
H	1.856718000	-1.534863000	0.375046000
C	3.496422000	-0.214054000	-0.165157000
C	4.406797000	-0.979908000	0.578988000
C	3.975758000	0.915167000	-0.840872000
C	5.743215000	-0.627103000	0.642854000
H	4.060382000	-1.862171000	1.110973000
C	5.316551000	1.269892000	-0.776241000
H	3.300137000	1.527668000	-1.426897000
C	6.207500000	0.503325000	-0.035582000
H	5.690724000	2.143186000	-1.299433000
O	7.519555000	0.853447000	0.025218000
H	7.992069000	0.209526000	0.573823000
O	6.697552000	-1.316392000	1.344450000
H	6.324114000	-2.095987000	1.774058000
C	1.071972000	-0.057953000	-0.836979000
H	1.205299000	0.839284000	-1.434381000
C	-0.300655000	-0.630033000	-0.768204000
O	-0.528031000	-1.652275000	-0.105353000
C	-1.337538000	0.122809000	-1.457048000
C	-2.615098000	-0.456915000	-1.850238000
O	-3.447691000	0.222193000	-2.463490000
C	-2.962948000	-1.908840000	-1.541013000
C	-3.346284000	-2.277772000	-0.121367000
O	-3.277011000	-3.416690000	0.285664000
H	-3.823409000	-2.163157000	-2.168616000

H	-2.127773000	-2.562923000	-1.796077000
H	-0.982167000	0.847085000	-2.191686000
S	-4.024117000	-0.969962000	0.877438000
C	-4.376698000	-1.895471000	2.391973000
H	-5.093242000	-2.692228000	2.195012000
H	-3.458904000	-2.319624000	2.798220000
H	-4.800747000	-1.182956000	3.100264000
C	-1.944036000	2.697130000	1.256168000
C	-2.829588000	3.691451000	1.545933000
N	-3.741131000	3.696464000	0.513299000
C	-3.403260000	2.733197000	-0.360073000
N	-2.317771000	2.116791000	0.067249000
H	-2.892225000	4.379335000	2.373249000
H	-3.939601000	2.490664000	-1.264935000
H	-1.080841000	2.360957000	1.808826000
H	-1.792398000	1.122586000	-0.561607000
H	-4.535690000	4.314357000	0.423046000

$IM_{1.3.1}$

C	2.177059000	-0.759007000	-0.269652000
H	1.961923000	-1.721227000	0.191551000
C	3.568419000	-0.301679000	-0.187509000
C	4.491960000	-1.099795000	0.503906000
C	4.025987000	0.890991000	-0.761719000
C	5.816932000	-0.715711000	0.618716000
H	4.164914000	-2.032474000	0.956281000
C	5.355518000	1.276547000	-0.647115000
H	3.341934000	1.528581000	-1.310150000
C	6.258323000	0.478282000	0.043282000
H	5.711277000	2.198844000	-1.093761000
O	7.560485000	0.859012000	0.154194000
H	8.042494000	0.184296000	0.655181000
O	6.780058000	-1.435350000	1.278150000
H	6.419762000	-2.255156000	1.638418000
C	1.140356000	-0.136019000	-0.843323000
H	1.254887000	0.828942000	-1.330120000
C	-0.225632000	-0.742423000	-0.851850000
O	-0.413340000	-1.858742000	-0.322527000
C	-1.242661000	0.048736000	-1.468030000
C	-2.568929000	-0.364721000	-1.738531000
O	-3.437079000	0.420965000	-2.199761000
C	-2.990902000	-1.812610000	-1.510050000
C	-3.337743000	-2.242603000	-0.100510000
O	-3.370190000	-3.407156000	0.231257000

H	-3.893781000	-1.975211000	-2.108593000
H	-2.211875000	-2.502564000	-1.836265000
H	-0.948515000	0.999119000	-1.907810000
S	-3.814924000	-0.948595000	1.034072000
C	-4.178198000	-1.947363000	2.498511000
H	-4.992501000	-2.642285000	2.295536000
H	-3.292520000	-2.501955000	2.806831000
H	-4.472491000	-1.252349000	3.285759000
C	-2.286879000	3.039976000	1.259336000
C	-3.264707000	3.976921000	1.361481000
N	-4.127114000	3.754497000	0.306814000
C	-3.694530000	2.720074000	-0.414119000
N	-2.579587000	2.278885000	0.150587000
H	-3.420814000	4.767000000	2.076916000
H	-4.138971000	2.278884000	-1.294760000
H	-1.422002000	2.851969000	1.873856000
H	-2.042526000	1.469934000	-0.210395000
H	-4.963882000	4.285486000	0.104059000

$TS2_{1.3.1}$

C	1.835696000	-0.537681000	-0.418659000
H	1.687567000	-1.495531000	0.074505000
C	3.187418000	0.020238000	-0.336735000
C	4.170614000	-0.720468000	0.337055000
C	3.551283000	1.253480000	-0.892307000
C	5.464816000	-0.243993000	0.449000000
H	3.915396000	-1.682089000	0.774448000
C	4.849973000	1.731902000	-0.779865000
H	2.818079000	1.852672000	-1.420025000
C	5.813648000	0.988655000	-0.110090000
H	5.134268000	2.686123000	-1.210098000
O	7.083991000	1.460673000	-0.001461000
H	7.618771000	0.815252000	0.484654000
O	6.483083000	-0.900688000	1.089024000
H	6.187982000	-1.749243000	1.442277000
C	0.769726000	-0.003638000	-1.033373000
H	0.828445000	0.943899000	-1.560433000
C	-0.542875000	-0.663144000	-1.051952000
O	-0.633892000	-1.813794000	-0.458912000
C	-1.610682000	-0.035397000	-1.670553000
C	-2.883996000	-0.663016000	-1.777282000
O	-3.901499000	-0.075826000	-2.200138000
C	-2.971401000	-2.122941000	-1.380365000
C	-2.187061000	-2.513141000	-0.135926000

O	-1.985727000	-3.688964000	0.164553000
H	-4.019358000	-2.394968000	-1.234687000
H	-2.573309000	-2.733154000	-2.199354000
H	-1.479201000	0.954472000	-2.091866000
S	-2.802190000	-1.396433000	1.330663000
C	-1.736940000	-2.059654000	2.633227000
H	-1.580528000	-3.116734000	2.406911000
H	-0.777373000	-1.542841000	2.668804000
H	-2.248525000	-1.961802000	3.591259000
C	-3.162715000	2.838254000	1.781684000
C	-3.699515000	3.927419000	1.174175000
N	-4.089234000	3.515629000	-0.083953000
C	-3.803344000	2.223443000	-0.244473000
N	-3.238437000	1.800477000	0.879497000
H	-3.836165000	4.938806000	1.518752000
H	-3.985011000	1.609290000	-1.125858000
H	-2.738606000	2.715994000	2.764589000
H	-2.930006000	0.822762000	1.038266000
H	-4.529217000	4.094162000	-0.787464000

$P_{1.3.1}$

C	2.460287000	-0.553909000	-0.169197000
H	2.317856000	-1.243353000	0.658366000
C	3.791274000	0.038512000	-0.272309000
C	4.747202000	-0.313387000	0.694282000
C	4.164009000	0.934316000	-1.283049000
C	6.024515000	0.213922000	0.649268000
H	4.481045000	-1.005397000	1.488745000
C	5.446158000	1.462841000	-1.328357000
H	3.452285000	1.227480000	-2.046101000
C	6.383004000	1.107521000	-0.365536000
H	5.738666000	2.156837000	-2.108655000
O	7.636022000	1.626392000	-0.413784000
H	8.151562000	1.277390000	0.329005000
O	7.018639000	-0.071434000	1.546493000
H	6.713952000	-0.679101000	2.231923000
C	1.411718000	-0.331884000	-0.982114000
H	1.475507000	0.350919000	-1.822415000
C	0.121143000	-0.964519000	-0.813258000
O	0.081046000	-1.846604000	0.229866000
C	-0.951299000	-0.701358000	-1.597075000
C	-2.216913000	-1.349932000	-1.376011000
O	-3.238472000	-1.081824000	-2.009784000
C	-2.228764000	-2.465847000	-0.359240000

C	-1.054033000	-2.534886000	0.573850000
O	-1.008197000	-3.227348000	1.553996000
H	-3.140284000	-2.428011000	0.236548000
H	-2.246421000	-3.414853000	-0.912687000
H	-0.869968000	0.034900000	-2.386463000
S	-2.472469000	0.404403000	1.725700000
C	-1.628987000	1.924118000	1.140308000
H	-1.203525000	2.482427000	1.977742000
H	-0.816991000	1.677010000	0.451169000
H	-2.325147000	2.586502000	0.614870000
C	-6.114603000	1.842019000	1.030634000
C	-7.111464000	1.901540000	0.106512000
N	-6.678452000	1.154644000	-0.968540000
C	-5.461961000	0.663181000	-0.699173000
N	-5.105094000	1.070140000	0.506592000
H	-8.068758000	2.396161000	0.116389000
H	-4.864030000	0.035806000	-1.349174000
H	-6.042967000	2.281833000	2.012395000
H	-4.119211000	0.796163000	1.005591000
H	-7.189255000	0.996418000	-1.826323000

$R_{1.3.2}$

C	-1.420447000	0.117399000	-0.064266000
H	-1.126753000	1.158296000	-0.171646000
C	-2.861401000	-0.124814000	0.014544000
C	-3.721783000	0.982911000	-0.039023000
C	-3.427789000	-1.399770000	0.139723000
C	-5.093447000	0.818634000	0.031904000
H	-3.308636000	1.983238000	-0.137383000
C	-4.804390000	-1.564453000	0.210722000
H	-2.795403000	-2.279144000	0.181825000
C	-5.645138000	-0.459781000	0.157929000
H	-5.245480000	-2.550503000	0.307435000
O	-6.991736000	-0.625852000	0.228054000
H	-7.419625000	0.242060000	0.177083000
O	-6.003108000	1.841987000	-0.012532000
H	-5.566877000	2.698980000	-0.097698000
C	-0.442409000	-0.801795000	-0.017409000
H	-0.658324000	-1.859727000	0.089818000
C	0.970308000	-0.478800000	-0.104759000
O	1.242548000	0.840812000	-0.234733000
C	1.966597000	-1.396778000	-0.063535000
C	3.321406000	-0.953096000	-0.163615000
O	4.253534000	-1.899554000	-0.114954000

C	3.593925000	0.385099000	-0.301032000
C	2.539113000	1.341791000	-0.336513000
O	2.637994000	2.552698000	-0.444090000
H	6.989730000	1.985316000	1.064262000
H	4.605040000	0.757592000	-0.389100000
H	1.747515000	-2.450798000	0.041976000
O	6.733443000	-1.025405000	-0.278287000
H	5.176184000	-1.529127000	-0.185567000
H	7.188260000	-1.061131000	-1.127892000
O	7.573954000	1.217070000	1.026108000
H	7.945558000	1.112790000	1.911156000
H	7.048517000	-0.221173000	0.191188000

$TS_{1.3.2}$

C	-1.211023000	0.149587000	-0.163072000
H	-0.953609000	1.205404000	-0.172211000
C	-2.637843000	-0.149470000	-0.041887000
C	-3.533526000	0.927323000	0.052849000
C	-3.156948000	-1.450219000	-0.014861000
C	-4.893891000	0.707576000	0.172085000
H	-3.156712000	1.946563000	0.032155000
C	-4.522261000	-1.670405000	0.104063000
H	-2.495859000	-2.306140000	-0.087187000
C	-5.398330000	-0.596288000	0.198355000
H	-4.927102000	-2.676300000	0.125099000
O	-6.733443000	-0.815970000	0.315124000
H	-7.190219000	0.037086000	0.367581000
O	-5.836807000	1.696057000	0.270977000
H	-5.432901000	2.572643000	0.253702000
C	-0.205579000	-0.735780000	-0.262851000
H	-0.385082000	-1.805708000	-0.262174000
C	1.191271000	-0.356856000	-0.381343000
O	1.405067000	0.984901000	-0.372572000
C	2.205490000	-1.247429000	-0.488472000
C	3.573667000	-0.793340000	-0.587091000
O	4.519788000	-1.622591000	-0.677246000
C	3.788693000	0.628097000	-0.500139000
C	2.675072000	1.535866000	-0.550053000
O	2.710803000	2.743471000	-0.679922000
H	4.422363000	0.746167000	0.902379000
H	4.695347000	1.019330000	-0.957367000
H	1.999203000	-2.309840000	-0.502516000
O	6.567077000	-1.011447000	0.732725000
H	5.883150000	-1.306655000	0.043223000

H	7.386477000	-0.763392000	0.286626000
O	5.155539000	0.714412000	1.752767000
H	4.733546000	0.451775000	2.585238000
H	5.868465000	-0.013577000	1.425129000

$P_{1.3.2}$

C	-1.292975000	0.125072000	-0.162541000
H	-1.030079000	1.176601000	-0.239425000
C	-2.717774000	-0.156292000	-0.028031000
C	-3.603372000	0.932944000	0.025663000
C	-3.246818000	-1.451627000	0.050537000
C	-4.963999000	0.730261000	0.158627000
H	-3.217171000	1.946767000	-0.036093000
C	-4.612631000	-1.654690000	0.182087000
H	-2.593530000	-2.315327000	0.007413000
C	-5.478535000	-0.568837000	0.237907000
H	-5.027052000	-2.654888000	0.242934000
O	-6.812351000	-0.772206000	0.367668000
H	-7.263444000	0.085413000	0.392409000
O	-5.899597000	1.727073000	0.221433000
H	-5.491252000	2.600953000	0.180491000
C	-0.289705000	-0.772567000	-0.196856000
H	-0.472868000	-1.838557000	-0.120217000
C	1.101377000	-0.406973000	-0.329620000
O	1.304300000	0.943345000	-0.426240000
C	2.116531000	-1.305647000	-0.368642000
C	3.485007000	-0.884457000	-0.474713000
O	4.426834000	-1.679612000	-0.543717000
C	3.744590000	0.601087000	-0.459098000
C	2.549566000	1.496163000	-0.595786000
O	2.603712000	2.675889000	-0.812126000
H	4.237663000	0.851501000	0.495592000
H	4.464858000	0.869530000	-1.235713000
H	1.897028000	-2.363953000	-0.313647000
O	6.932675000	-1.059248000	0.381627000
H	6.103383000	-1.326017000	-0.072168000
H	7.599687000	-0.919651000	-0.300187000
O	5.896207000	0.993139000	1.875422000
H	6.032490000	0.875101000	2.821964000
H	6.398485000	0.278875000	1.426910000

$R_{2.1}$

N	1.407848000	-3.755626000	-1.044924000
C	0.084743000	-3.944026000	-0.924260000

O	-0.538350000	-4.928340000	-1.351476000
N	-0.652020000	-2.954586000	-0.243532000
C	-0.171561000	-1.785069000	0.291252000
O	-0.892139000	-0.998614000	0.912054000
C	1.244177000	-1.590729000	0.051766000
N	1.872689000	-0.450166000	0.486978000
C	3.148689000	-0.148001000	-0.027440000
C	3.800624000	0.905508000	0.804285000
C	5.108803000	0.876328000	1.114374000
C	5.738093000	1.983284000	1.917314000
C	5.920578000	-0.255160000	0.682412000
C	7.393458000	-0.241964000	0.951534000
C	5.352226000	-1.348656000	0.084989000
C	3.962849000	-1.407889000	-0.164964000
N	3.343040000	-2.511311000	-0.576146000
C	1.934482000	-2.622518000	-0.553333000
C	4.126067000	-3.703437000	-0.936422000
C	0.271156000	2.876147000	0.502909000
C	0.628425000	4.046747000	-0.162947000
O	2.785332000	1.659695000	-1.552958000
C	-0.299506000	4.815339000	-0.920606000
O	-0.013840000	5.884637000	-1.514179000
C	-1.653952000	4.264501000	-0.979086000
C	-1.963254000	3.115318000	-0.342251000
H	3.146914000	1.715797000	1.108520000
H	6.174964000	1.608288000	2.848085000
H	6.538200000	2.476954000	1.357232000
H	4.992333000	2.737188000	2.172683000
H	7.594714000	-0.152873000	2.023868000
H	7.875400000	-1.147998000	0.582394000
H	7.863587000	0.622005000	0.470606000
H	5.976287000	-2.200028000	-0.149091000
H	4.892340000	-3.425642000	-1.659072000
H	3.437822000	-4.420292000	-1.371363000
H	1.812684000	1.674382000	-1.521227000
H	-2.408588000	4.786398000	-1.555853000
O	-1.044618000	2.437607000	0.389658000
O	0.992479000	2.131584000	1.200453000
C	-3.270826000	2.472020000	-0.371371000
H	-4.045087000	3.032223000	-0.887073000
C	-3.507754000	1.270053000	0.176321000
H	-2.673224000	0.755438000	0.646274000
C	-4.782841000	0.546795000	0.198829000
C	-4.788390000	-0.749183000	0.736600000

C	-5.990635000	1.072900000	-0.275508000
C	-5.957442000	-1.487832000	0.791331000
H	-3.859187000	-1.172797000	1.109809000
C	-7.163376000	0.329521000	-0.221990000
H	-6.025335000	2.075336000	-0.687264000
C	-7.154427000	-0.953640000	0.308734000
H	-8.100180000	0.735898000	-0.588244000
O	-8.306223000	-1.680593000	0.361630000
H	-8.110072000	-2.544612000	0.753325000
O	-6.050226000	-2.758399000	1.301383000
H	-5.192604000	-3.071583000	1.614674000
H	4.585795000	-4.127443000	-0.041531000
H	1.315612000	0.327928000	0.874856000
H	-1.643052000	-3.131955000	-0.140172000
O	3.109373000	0.270640000	-1.451667000
H	1.653049000	4.385334000	-0.072099000

$TS_{2.1}$

N	0.912286000	-3.663114000	-1.247553000
C	-0.409613000	-3.807591000	-1.072851000
O	-1.106206000	-4.697930000	-1.590210000
N	-1.059018000	-2.887249000	-0.226201000
C	-0.484409000	-1.825678000	0.427652000
O	-1.129211000	-1.084880000	1.184357000
C	0.921165000	-1.678056000	0.149736000
N	1.639049000	-0.651901000	0.725593000
C	2.866007000	-0.270649000	0.087839000
C	3.622908000	0.621558000	1.031178000
C	4.942936000	0.504321000	1.254992000
C	5.672964000	1.473546000	2.147573000
C	5.673908000	-0.590041000	0.624301000
C	7.159110000	-0.669987000	0.802396000
C	5.028311000	-1.563989000	-0.087749000
C	3.621206000	-1.542964000	-0.245215000
N	2.935932000	-2.566320000	-0.747908000
C	1.523673000	-2.633134000	-0.636426000
C	3.641749000	-3.717135000	-1.328940000
C	0.605805000	3.096726000	0.787340000
C	1.271330000	3.758117000	-0.289842000
O	2.043774000	1.902349000	-0.819674000
C	0.542418000	4.244127000	-1.435278000
O	1.043448000	4.995555000	-2.296561000
C	-0.823184000	3.747464000	-1.548164000
C	-1.368187000	2.977920000	-0.580179000

H	3.044061000	1.424793000	1.476787000
H	6.145336000	0.967108000	2.995332000
H	6.461634000	2.003703000	1.604417000
H	4.980185000	2.218119000	2.542773000
H	7.423021000	-0.735899000	1.862775000
H	7.577710000	-1.534970000	0.286380000
H	7.641474000	0.232926000	0.413913000
H	5.603115000	-2.390667000	-0.483052000
H	4.350020000	-3.366281000	-2.079044000
H	2.894093000	-4.355362000	-1.788335000
H	1.627889000	1.907964000	-1.697488000
H	-1.401286000	3.997338000	-2.429429000
O	-0.692199000	2.663473000	0.559063000
O	1.078010000	2.803297000	1.877395000
C	-2.701668000	2.400526000	-0.644985000
H	-3.316796000	2.755305000	-1.466270000
C	-3.131479000	1.454780000	0.205100000
H	-2.434801000	1.092282000	0.955059000
C	-4.444620000	0.806017000	0.203731000
C	-4.599052000	-0.359234000	0.970430000
C	-5.549509000	1.289194000	-0.507237000
C	-5.813954000	-1.019318000	1.009563000
H	-3.744735000	-0.752067000	1.516511000
C	-6.769423000	0.625027000	-0.465272000
H	-5.467228000	2.200321000	-1.089458000
C	-6.908599000	-0.531756000	0.290249000
H	-7.629386000	0.998077000	-1.011204000
O	-8.105893000	-1.179770000	0.333508000
H	-8.014920000	-1.963461000	0.895720000
O	-6.051545000	-2.164500000	1.726987000
H	-5.256764000	-2.453612000	2.192659000
H	4.165927000	-4.267749000	-0.545063000
H	1.093253000	0.082611000	1.164593000
H	-2.053062000	-3.027689000	-0.097072000
O	2.738073000	0.312591000	-1.172933000
H	2.228768000	4.207064000	-0.063136000

$P_{2.1}$

N	-0.229753000	-3.039944000	1.545274000
C	1.078846000	-3.090350000	1.263415000
O	1.926233000	-3.738772000	1.906262000
N	1.531099000	-2.363610000	0.146149000
C	0.767274000	-1.583741000	-0.687192000
O	1.258848000	-0.992272000	-1.665059000

C	-0.619653000	-1.531481000	-0.317282000
N	-1.515034000	-0.793577000	-1.070245000
C	-2.712711000	-0.334313000	-0.378117000
C	-3.671743000	0.148537000	-1.434974000
C	-4.978132000	-0.169409000	-1.469622000
C	-5.913135000	0.449091000	-2.476913000
C	-5.493505000	-1.120178000	-0.490144000
C	-6.964052000	-1.409985000	-0.463984000
C	-4.665063000	-1.764557000	0.386374000
C	-3.262128000	-1.551875000	0.355625000
N	-2.413935000	-2.318860000	1.034831000
C	-1.021586000	-2.282827000	0.762542000
C	-2.908629000	-3.298812000	2.011194000
C	-0.934248000	2.730704000	-0.769197000
C	-1.773384000	3.469707000	0.274965000
O	-3.093189000	3.058169000	0.229093000
C	-1.172317000	3.455692000	1.688292000
O	-1.856631000	3.788564000	2.645957000
C	0.224874000	3.100600000	1.803832000
C	0.918028000	2.594812000	0.761606000
H	-3.259132000	0.886130000	-2.117880000
H	-6.396644000	-0.307769000	-3.103262000
H	-6.707567000	1.024492000	-1.990530000
H	-5.365015000	1.127930000	-3.132701000
H	-7.304247000	-1.798618000	-1.429307000
H	-7.213926000	-2.137190000	0.309877000
H	-7.533736000	-0.494152000	-0.275100000
H	-5.089881000	-2.489775000	1.067902000
H	-3.586826000	-2.805732000	2.707142000
H	-2.048257000	-3.691486000	2.542996000
H	-3.045054000	2.045383000	0.366883000
H	0.697875000	3.171486000	2.775326000
O	0.369397000	2.436857000	-0.485285000
O	-1.329918000	2.451729000	-1.870225000
C	2.291518000	2.139787000	0.837246000
H	2.797112000	2.355764000	1.772676000
C	2.881391000	1.439138000	-0.146954000
H	2.286941000	1.190887000	-1.021024000
C	4.243780000	0.910046000	-0.149882000
C	4.566239000	-0.048090000	-1.124170000
C	5.235315000	1.308482000	0.754549000
C	5.835247000	-0.593842000	-1.177697000
H	3.796653000	-0.376732000	-1.818467000
C	6.510808000	0.762014000	0.695659000

H	5.019396000	2.060358000	1.505450000
C	6.817470000	-0.191224000	-0.267180000
H	7.284551000	1.067889000	1.391484000
O	8.067358000	-0.723951000	-0.324702000
H	8.092849000	-1.384046000	-1.033618000
O	6.237442000	-1.537301000	-2.087623000
H	5.505860000	-1.805308000	-2.657977000
H	-3.425121000	-4.113085000	1.498072000
H	-1.076777000	-0.111147000	-1.680963000
H	2.522185000	-2.420298000	-0.051307000
O	-2.519536000	0.614879000	0.572497000
H	-1.720939000	4.526158000	-0.036973000

$R_{2.2}$

C	-2.349358000	1.517568000	-0.335909000
C	-3.523010000	0.592302000	-0.118920000
O	-4.700004000	1.129938000	-0.647322000
C	-3.278757000	-0.816613000	-0.634865000
O	-4.216803000	-1.536539000	-0.961598000
C	-1.904059000	-1.244080000	-0.605205000
C	-0.891180000	-0.370489000	-0.386784000
H	-4.669820000	2.090872000	-0.526923000
H	-1.679817000	-2.288507000	-0.778922000
O	-1.103278000	0.982093000	-0.241219000
O	-2.471456000	2.700060000	-0.509185000
C	0.500633000	-0.742795000	-0.320042000
H	0.690135000	-1.804285000	-0.432931000
C	1.499161000	0.141114000	-0.129063000
H	1.232220000	1.189330000	-0.025682000
C	2.924542000	-0.152972000	-0.044128000
C	3.809961000	0.920198000	0.152688000
C	3.454460000	-1.446254000	-0.148617000
C	5.171911000	0.703176000	0.240953000
H	3.422538000	1.932112000	0.235818000
C	4.821311000	-1.663468000	-0.059781000
H	2.800901000	-2.297339000	-0.300481000
C	5.687428000	-0.593892000	0.134967000
H	5.236434000	-2.661999000	-0.139717000
O	7.022128000	-0.810734000	0.221214000
H	7.473916000	0.036442000	0.354915000
O	6.108746000	1.682093000	0.431487000
H	5.702887000	2.555799000	0.493048000
H	-3.605910000	0.464295000	0.979650000
O	-5.057972000	-0.273009000	2.476581000

H	-5.135149000	-0.845935000	3.247392000
H	-5.709910000	-0.597871000	1.817315000
O	-6.630817000	-1.048030000	0.336997000
H	-5.872885000	-1.211804000	-0.262075000
H	-7.028157000	-0.230865000	0.011189000

$TS_{2.2}$

C	-2.457304000	1.463900000	-0.365061000
C	-3.560096000	0.540610000	-0.303732000
O	-4.799837000	1.085400000	-0.660851000
C	-3.341004000	-0.849319000	-0.633052000
O	-4.280367000	-1.650867000	-0.841462000
C	-1.959250000	-1.280143000	-0.573020000
C	-0.953854000	-0.394043000	-0.386755000
H	-4.678355000	2.047778000	-0.692407000
H	-1.737598000	-2.331639000	-0.703443000
O	-1.190046000	0.943985000	-0.251353000
O	-2.576732000	2.673787000	-0.448944000
C	0.448605000	-0.755269000	-0.309227000
H	0.646452000	-1.816948000	-0.411493000
C	1.439749000	0.134254000	-0.130405000
H	1.166576000	1.182487000	-0.042691000
C	2.871442000	-0.151162000	-0.043198000
C	3.753896000	0.931033000	0.102162000
C	3.407767000	-1.444110000	-0.096374000
C	5.118601000	0.724013000	0.188969000
H	3.363608000	1.944423000	0.145031000
C	4.777346000	-1.651559000	-0.008619000
H	2.756875000	-2.304042000	-0.204718000
C	5.640500000	-0.572232000	0.133780000
H	5.195575000	-2.651361000	-0.048508000
O	6.979560000	-0.779511000	0.219671000
H	7.426276000	0.075423000	0.312925000
O	6.049760000	1.718028000	0.331109000
H	5.634984000	2.589102000	0.363509000
H	-3.859560000	0.316486000	1.134093000
O	-4.536397000	0.121431000	2.061857000
H	-4.079504000	-0.404349000	2.735691000
H	-5.344576000	-0.418413000	1.654614000
O	-6.264932000	-1.164448000	0.813802000
H	-5.672803000	-1.374199000	0.034855000
H	-6.984407000	-0.613018000	0.479098000

$P_{2.2}$

C	-2.271247000	1.405699000	-0.291089000
C	-3.354964000	0.477794000	-0.260644000
O	-4.609636000	1.032706000	-0.327535000
C	-3.132288000	-0.871661000	-0.195993000
O	-4.063417000	-1.818145000	-0.193469000
C	-1.774302000	-1.318189000	-0.118767000
C	-0.753353000	-0.429229000	-0.130672000
H	-4.471195000	1.977410000	-0.524910000
H	-1.576726000	-2.380048000	-0.054470000
O	-1.004715000	0.906550000	-0.219373000
O	-2.430715000	2.617827000	-0.389447000
C	0.652361000	-0.776256000	-0.056326000
H	0.847867000	-1.841228000	0.014779000
C	1.648928000	0.124056000	-0.071053000
H	1.379277000	1.174618000	-0.141485000
C	3.084629000	-0.150831000	0.001084000
C	3.967326000	0.940510000	0.000483000
C	3.625247000	-1.441056000	0.071321000
C	5.335052000	0.745849000	0.071479000
H	3.575137000	1.952437000	-0.056099000
C	4.998013000	-1.636232000	0.141014000
H	2.975853000	-2.308952000	0.070514000
C	5.860849000	-0.547542000	0.142501000
H	5.418798000	-2.634345000	0.195169000
O	7.203570000	-0.743400000	0.212072000
H	7.648543000	0.117351000	0.204565000
O	6.264663000	1.752048000	0.077772000
H	5.844988000	2.620883000	0.046124000
H	-5.963194000	0.989995000	0.983417000
O	-6.848385000	0.741341000	1.308458000
H	-6.814032000	0.734214000	2.272313000
H	-6.921693000	-0.726085000	0.369035000
O	-6.652713000	-1.478682000	-0.206002000
H	-5.017360000	-1.521324000	-0.251469000
H	-7.112974000	-1.374358000	-1.047314000

$R_{3.1}$

C	-4.185148000	-2.038605000	0.038946000
C	-3.612160000	-0.637354000	-0.212301000
O	-3.346080000	-3.020646000	0.251671000
O	-5.388077000	-2.193732000	0.033627000
O	-4.597482000	0.216159000	-0.420358000
C	-1.081786000	-0.975109000	-0.038662000
C	0.157518000	-0.198473000	-0.126622000

H	0.063668000	0.862845000	-0.327424000
C	1.354541000	-0.790815000	0.038624000
H	1.341051000	-1.860479000	0.237259000
C	2.672131000	-0.170393000	-0.014017000
C	3.796553000	-0.985643000	0.197628000
C	2.872655000	1.194285000	-0.262638000
C	5.070931000	-0.452082000	0.164954000
H	3.664485000	-2.047253000	0.387764000
C	4.151635000	1.729321000	-0.297743000
H	2.026438000	1.850123000	-0.431634000
C	5.256382000	0.912834000	-0.083918000
H	4.313587000	2.784169000	-0.489907000
O	6.504005000	1.440611000	-0.117281000
H	7.150610000	0.738483000	0.051967000
O	6.222876000	-1.164762000	0.360903000
H	6.040084000	-2.094872000	0.544409000
C	-2.308505000	-0.228612000	-0.234994000
H	-2.167220000	0.829307000	-0.432083000
H	-4.277098000	1.150752000	-0.581042000
O	-1.034561000	-2.206893000	0.193734000
H	-2.349062000	-2.733989000	0.234983000
O	-3.817862000	2.690252000	-0.800559000
H	-3.985165000	3.061553000	-1.675655000
H	-4.283245000	4.718122000	1.745063000
O	-4.859915000	4.383777000	1.046505000
H	-4.191497000	3.321360000	-0.142542000
H	-5.678370000	4.105624000	1.477355000

$TS_{3.1}$

C	-4.411939000	-1.518023000	-0.262172000
C	-3.773602000	-0.118972000	-0.553815000
O	-3.665939000	-2.446695000	0.280135000
O	-5.582497000	-1.684558000	-0.527698000
O	-4.576851000	0.727651000	-0.962162000
C	-1.247187000	-0.573911000	-0.015200000
C	0.068764000	0.059157000	-0.179118000
H	0.082534000	1.086381000	-0.526940000
C	1.198614000	-0.620585000	0.081125000
H	1.078008000	-1.647678000	0.419133000
C	2.573503000	-0.146529000	-0.039865000
C	3.612579000	-1.038084000	0.273719000
C	2.907206000	1.149831000	-0.453544000
C	4.933989000	-0.644390000	0.172338000
H	3.377094000	-2.048394000	0.598013000

C	4.233007000	1.545552000	-0.552636000
H	2.129223000	1.861993000	-0.702984000
C	5.252725000	0.653734000	-0.241811000
H	4.496752000	2.547391000	-0.873110000
O	6.547415000	1.043802000	-0.341841000
H	7.121902000	0.302202000	-0.097794000
O	6.011529000	-1.440455000	0.453998000
H	5.740363000	-2.334276000	0.697534000
C	-2.394088000	0.257353000	-0.311140000
H	-2.141485000	1.148893000	-0.885919000
H	-4.524601000	2.379276000	-0.471018000
O	-1.319836000	-1.753732000	0.410812000
H	-2.660188000	-2.186607000	0.375069000
O	-4.316475000	3.147794000	0.118938000
H	-5.137276000	3.617148000	0.313845000
H	-2.591036000	1.089148000	0.945579000
O	-2.840244000	1.914226000	1.694375000
H	-3.511282000	2.530424000	1.184185000
H	-3.238238000	1.559050000	2.504668000

$P_{3.1}$

C	-4.257710000	-1.404069000	0.012115000
C	-3.466932000	-0.150740000	-0.422997000
O	-3.558769000	-2.503937000	0.179392000
O	-5.456090000	-1.337411000	0.174831000
O	-3.850239000	0.458496000	-1.397642000
C	-1.017085000	-0.551577000	0.107559000
C	0.264274000	0.124543000	0.173694000
H	0.255367000	1.190745000	0.367350000
C	1.410718000	-0.570669000	0.004482000
H	1.303882000	-1.637360000	-0.182672000
C	2.770630000	-0.066937000	0.041236000
C	3.822053000	-0.981137000	-0.153429000
C	3.083380000	1.282913000	0.259759000
C	5.135106000	-0.555530000	-0.128067000
H	3.600095000	-2.030885000	-0.325664000
C	4.401018000	1.709760000	0.282826000
H	2.294664000	2.010205000	0.412274000
C	5.432659000	0.796708000	0.089618000
H	4.651813000	2.751202000	0.449898000
O	6.716675000	1.217589000	0.112504000
H	7.304709000	0.461024000	-0.036967000
O	6.226097000	-1.360328000	-0.304560000
H	5.974263000	-2.282547000	-0.438867000

C	-2.258860000	0.284505000	0.378106000
H	-2.072437000	1.336131000	0.157166000
H	-4.567599000	2.359153000	-1.339649000
O	-1.125850000	-1.763855000	-0.130475000
H	-2.572134000	-2.334521000	0.029475000
O	-4.939198000	3.035040000	-0.751619000
H	-5.681512000	3.433397000	-1.221524000
H	-2.490052000	0.203830000	1.447774000
O	-5.141400000	1.310446000	1.475761000
H	-5.199532000	2.005921000	0.793688000
H	-5.686837000	0.578766000	1.152770000

$R_{3.2}$

C	-3.804401000	-1.752779000	-0.344522000
C	-2.667412000	-2.591137000	0.309445000
O	-3.488129000	-0.952788000	-1.344776000
O	-4.941486000	-1.890263000	0.041410000
O	-2.922865000	-3.201792000	1.318245000
C	-0.495262000	-1.579097000	-0.738634000
C	0.885547000	-1.557540000	-0.281970000
H	1.221695000	-2.385129000	0.331429000
C	1.693084000	-0.528755000	-0.611772000
H	1.249878000	0.253956000	-1.224181000
C	3.085945000	-0.334235000	-0.244565000
C	3.735030000	0.826029000	-0.701395000
C	3.808725000	-1.243056000	0.541202000
C	5.056144000	1.066188000	-0.378297000
H	3.192884000	1.541243000	-1.313735000
C	5.135716000	-1.003754000	0.861983000
H	3.335108000	-2.145433000	0.909758000
C	5.766517000	0.148933000	0.406344000
H	5.701198000	-1.701017000	1.469967000
O	7.061776000	0.379574000	0.723598000
H	7.339493000	1.223630000	0.335647000
O	5.772885000	2.163280000	-0.771653000
H	5.229859000	2.783231000	-1.274446000
C	-1.330621000	-2.798883000	-0.392921000
H	-0.766253000	-3.491378000	0.230280000
O	-0.974346000	-0.681715000	-1.444364000
H	-2.487013000	-0.827131000	-1.463723000
H	-1.544932000	-3.304206000	-1.345344000
H	-2.362472000	-0.512701000	2.286905000
O	-1.970563000	-0.308074000	1.429551000
C	-2.898376000	3.442671000	1.112504000

C	-3.771865000	4.244948000	0.434245000
N	-4.426936000	3.411239000	-0.441640000
C	-3.939357000	2.161125000	-0.267439000
N	-3.011081000	2.145335000	0.665992000
H	-3.982019000	5.300825000	0.493374000
H	-4.275509000	1.302799000	-0.831869000
H	-2.200452000	3.716669000	1.889599000
H	-5.147559000	3.678019000	-1.096239000
H	-2.292455000	0.600895000	1.190775000

$TS1_{3.2}$

C	3.750276000	-2.088959000	0.890323000
C	2.981380000	-2.183830000	-0.441796000
O	3.240773000	-1.233694000	1.702223000
O	4.751630000	-2.774118000	1.053420000
O	3.560028000	-2.086889000	-1.505766000
C	0.759158000	-0.972471000	-0.107873000
C	-0.659193000	-0.897674000	-0.508325000
H	-0.902293000	-1.333331000	-1.470653000
C	-1.579867000	-0.342629000	0.288177000
H	-1.236149000	0.054571000	1.240150000
C	-3.015114000	-0.211991000	0.025512000
C	-3.824557000	0.335255000	1.032285000
C	-3.617316000	-0.598777000	-1.177539000
C	-5.186383000	0.485116000	0.840919000
H	-3.379863000	0.644603000	1.974211000
C	-4.984174000	-0.449981000	-1.369248000
H	-3.020099000	-1.017039000	-1.979673000
C	-5.776179000	0.091013000	-0.364169000
H	-5.454579000	-0.747180000	-2.300113000
O	-7.112334000	0.236696000	-0.556614000
H	-7.505282000	0.628362000	0.237925000
O	-6.050466000	1.008445000	1.765669000
H	-5.589447000	1.273354000	2.571422000
C	1.465691000	-2.296061000	-0.398135000
H	1.127709000	-2.692515000	-1.356854000
O	1.095580000	-0.347047000	0.975949000
H	2.052619000	-0.710599000	1.346504000
H	1.155315000	-2.985948000	0.394469000
H	2.055716000	-0.473615000	-2.052961000
O	1.466435000	0.039123000	-1.474936000
C	2.983907000	3.234775000	-1.091245000
C	3.883514000	3.942186000	-0.349466000
N	4.318141000	3.076431000	0.627411000

C	3.688230000	1.897773000	0.458668000
N	2.871445000	1.962893000	-0.575758000
H	4.244058000	4.955082000	-0.425467000
H	3.842186000	1.032975000	1.089702000
H	2.414247000	3.551688000	-1.951292000
H	4.994933000	3.280368000	1.348982000
H	2.044404000	0.861363000	-1.075998000

$IM_{3.2}$

C	3.520029000	-2.232578000	0.917340000
C	2.728119000	-2.261706000	-0.395331000
O	3.258593000	-1.179174000	1.588509000
O	4.309965000	-3.140156000	1.175489000
O	3.299321000	-2.199971000	-1.475023000
C	0.746231000	-0.689563000	-0.395086000
C	-0.727594000	-0.614452000	-0.690924000
H	-0.997413000	-0.955562000	-1.686607000
C	-1.636776000	-0.175552000	0.179960000
H	-1.276970000	0.143276000	1.154766000
C	-3.088734000	-0.066494000	-0.033386000
C	-3.882111000	0.393441000	1.026818000
C	-3.720920000	-0.392857000	-1.238474000
C	-5.253836000	0.520129000	0.884598000
H	-3.418173000	0.653830000	1.974804000
C	-5.097406000	-0.268221000	-1.380921000
H	-3.139667000	-0.747763000	-2.081963000
C	-5.872115000	0.188334000	-0.322956000
H	-5.587763000	-0.521372000	-2.314776000
O	-7.220367000	0.311324000	-0.466694000
H	-7.595827000	0.637709000	0.364509000
O	-6.098600000	0.963641000	1.869418000
H	-5.614248000	1.181772000	2.675275000
C	1.217969000	-2.169587000	-0.339013000
H	0.818775000	-2.703826000	-1.204602000
O	1.041288000	-0.017337000	0.776645000
H	1.861186000	-0.417427000	1.182003000
H	0.814638000	-2.612791000	0.573309000
H	1.999951000	-0.651071000	-1.966664000
O	1.432402000	-0.021973000	-1.489058000
C	3.493609000	3.163926000	-0.982176000
C	4.530903000	3.626951000	-0.237611000
N	4.829894000	2.631561000	0.669332000
C	4.007018000	1.598308000	0.489158000
N	3.189985000	1.906954000	-0.509457000

H	5.071360000	4.558204000	-0.271069000
H	3.994162000	0.664913000	1.045503000
H	2.951925000	3.617905000	-1.795399000
H	5.560166000	2.665017000	1.368809000
H	2.451615000	1.266278000	-0.863293000

$TS2_{3.2}$

C	-3.088474000	2.824636000	0.840250000
C	-2.512296000	2.511546000	-0.535325000
O	-2.747107000	1.971515000	1.796624000
O	-3.817216000	3.772748000	1.043419000
O	-3.319916000	2.049723000	-1.399668000
C	-0.964106000	0.387675000	-0.438715000
C	0.472183000	0.313335000	-0.792305000
H	0.702984000	0.460994000	-1.841381000
C	1.410354000	0.072017000	0.130551000
H	1.069522000	-0.056867000	1.155563000
C	2.857118000	-0.039308000	-0.084262000
C	3.677725000	-0.293561000	1.024780000
C	3.463348000	0.094460000	-1.339498000
C	5.049968000	-0.404911000	0.880822000
H	3.233996000	-0.402749000	2.011103000
C	4.839843000	-0.019493000	-1.484191000
H	2.860555000	0.290754000	-2.218797000
C	5.641398000	-0.269033000	-0.377795000
H	5.310542000	0.085728000	-2.455694000
O	6.989386000	-0.378712000	-0.523181000
H	7.387476000	-0.538358000	0.345541000
O	5.921896000	-0.651264000	1.909574000
H	5.463497000	-0.686247000	2.758164000
C	-1.128634000	2.523040000	-0.699591000
H	-0.743605000	2.567673000	-1.712582000
O	-1.370978000	0.106132000	0.730850000
H	-2.188829000	1.205334000	1.428211000
H	-0.499984000	2.932906000	0.083437000
H	-2.503148000	0.732967000	-1.636678000
O	-1.777308000	0.029757000	-1.507610000
C	-3.897877000	-3.095161000	-1.075261000
C	-4.411884000	-3.870324000	-0.085113000
N	-3.962271000	-3.329988000	1.101974000
C	-3.202524000	-2.264708000	0.845676000
N	-3.152745000	-2.109873000	-0.468550000
H	-5.046301000	-4.740307000	-0.118583000
H	-2.702218000	-1.624787000	1.555556000

H	-4.000882000	-3.160581000	-2.145799000
H	-4.173736000	-3.675979000	2.028679000
H	-2.631675000	-1.331902000	-0.930904000

$P_{3.2}$

C	-2.995447000	2.908279000	0.630100000
C	-3.208157000	2.429728000	-0.783404000
O	-2.474053000	2.029807000	1.479260000
O	-3.284804000	4.032027000	0.992958000
O	-3.482584000	1.097901000	-0.902124000
C	-0.664075000	-0.330315000	-0.503313000
C	0.776732000	-0.442445000	-0.832143000
H	1.001422000	-0.746272000	-1.848821000
C	1.726992000	-0.172081000	0.072711000
H	1.384381000	0.133057000	1.059367000
C	3.179009000	-0.235569000	-0.109875000
C	4.000874000	0.119091000	0.970607000
C	3.788078000	-0.632974000	-1.306504000
C	5.378978000	0.077542000	0.854576000
H	3.552260000	0.432377000	1.909746000
C	5.170861000	-0.675083000	-1.422870000
H	3.181690000	-0.913810000	-2.159911000
C	5.973927000	-0.320979000	-0.345954000
H	5.645893000	-0.981902000	-2.348351000
O	7.327127000	-0.363733000	-0.463322000
H	7.725025000	-0.080905000	0.373710000
O	6.255163000	0.404896000	1.856100000
H	5.789742000	0.689433000	2.652416000
C	-3.160168000	3.284412000	-1.802215000
H	-3.309109000	2.943091000	-2.820281000
O	-1.034456000	-0.019800000	0.659219000
H	-2.087123000	1.216546000	1.047389000
H	-2.965998000	4.333803000	-1.620653000
H	-2.725346000	0.569089000	-1.265258000
O	-1.492698000	-0.574743000	-1.455556000
C	-4.230025000	-3.024962000	-0.686987000
C	-4.953553000	-3.434633000	0.388033000
N	-4.488176000	-2.709408000	1.464978000
C	-3.518502000	-1.888152000	1.058683000
N	-3.348193000	-2.067660000	-0.241354000
H	-5.743195000	-4.161549000	0.480736000
H	-2.956678000	-1.194112000	1.663836000
H	-4.271604000	-3.330168000	-1.719533000
H	-4.825865000	-2.775913000	2.415951000

H	-2.643598000	-1.515276000	-0.806674000
